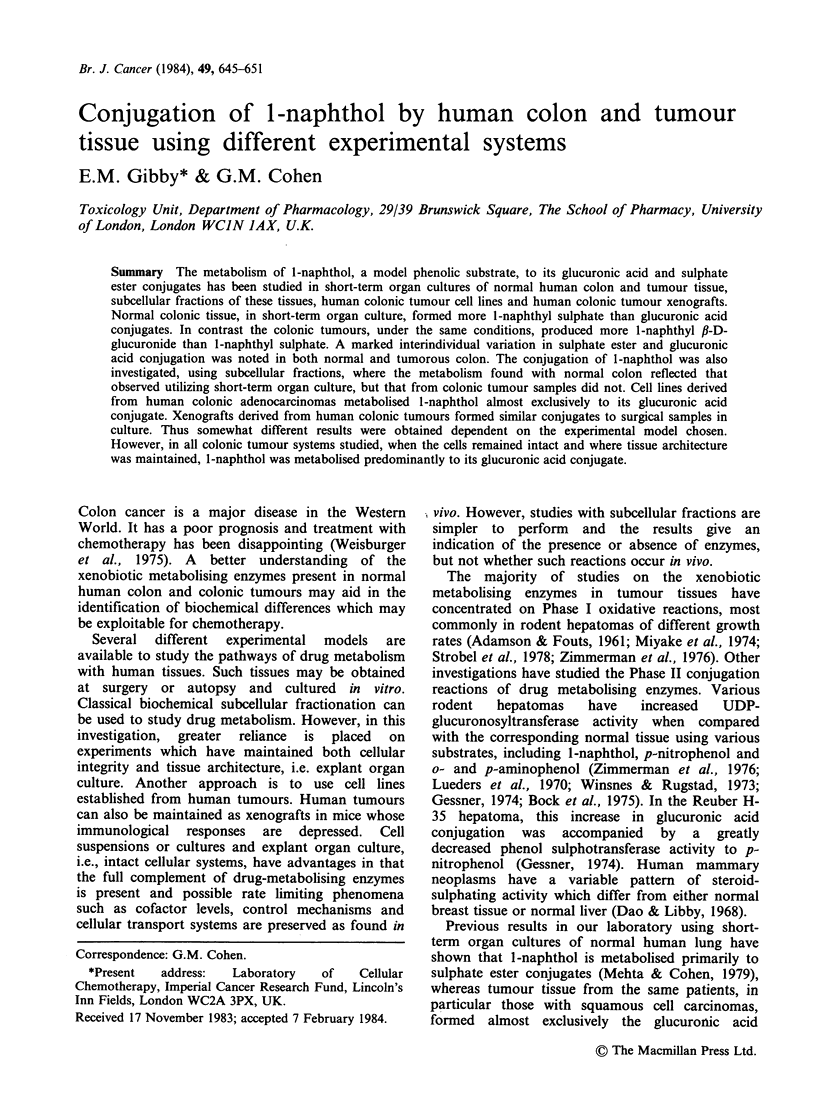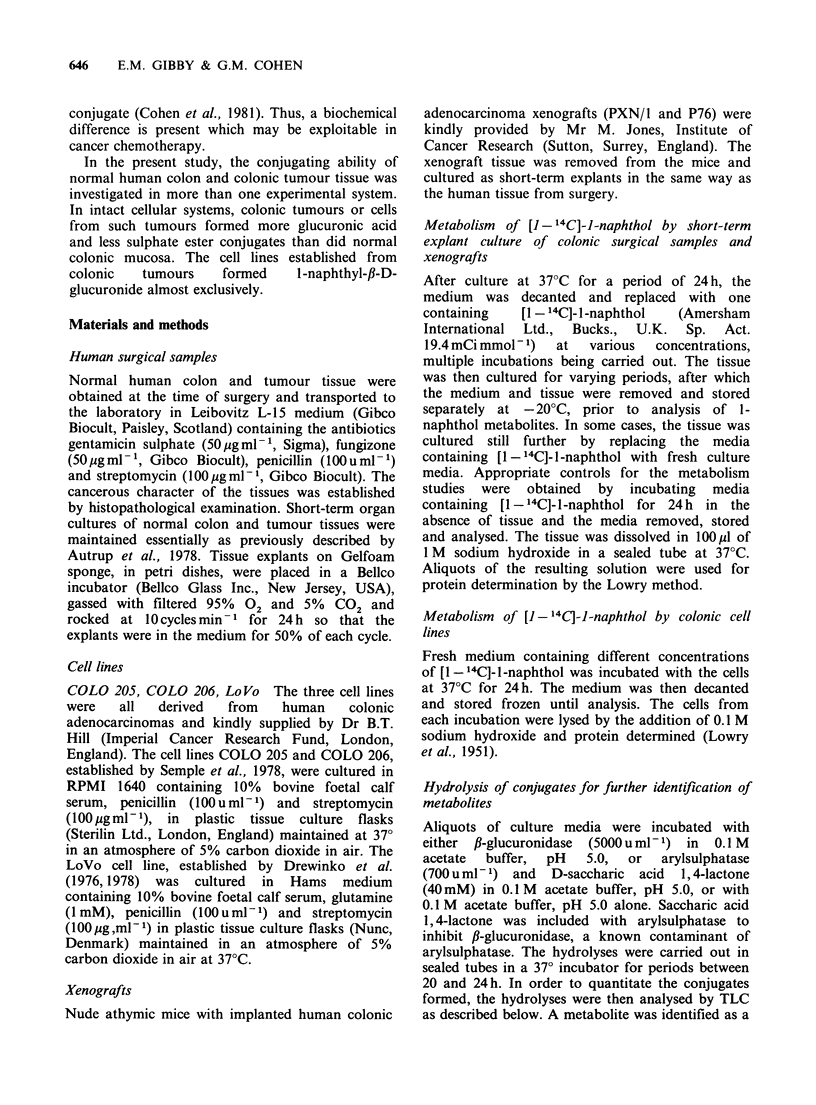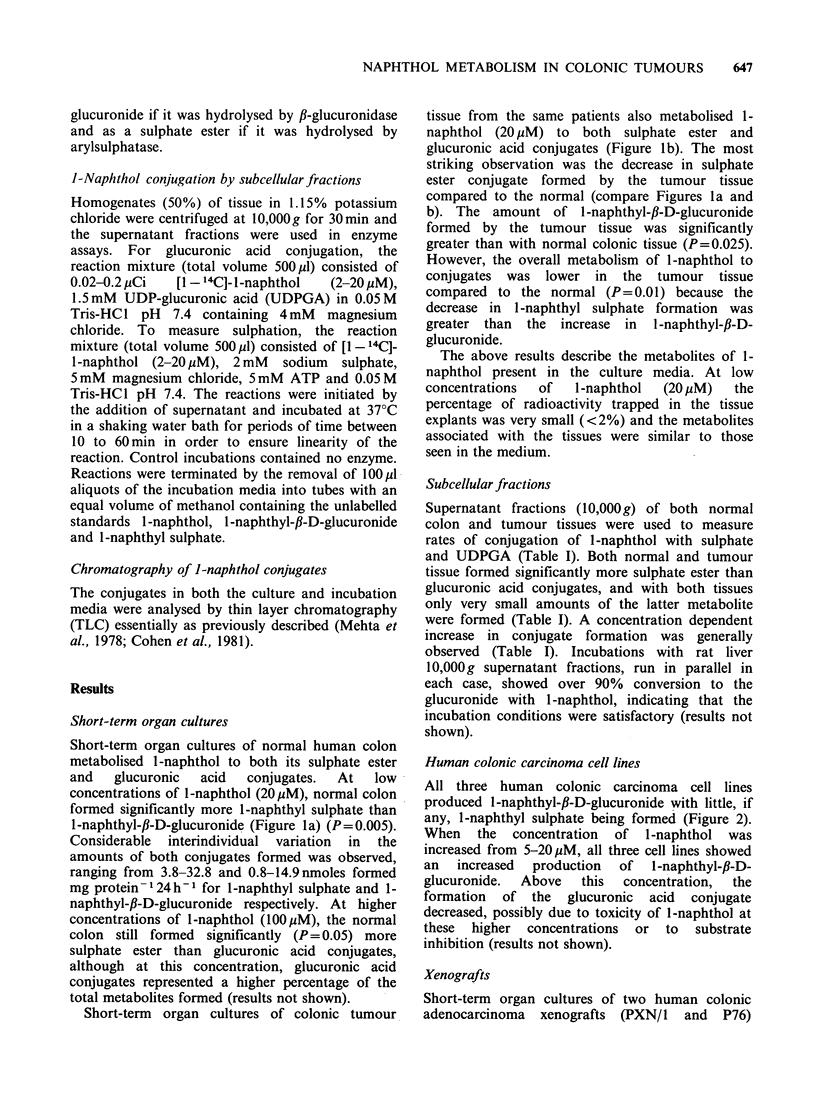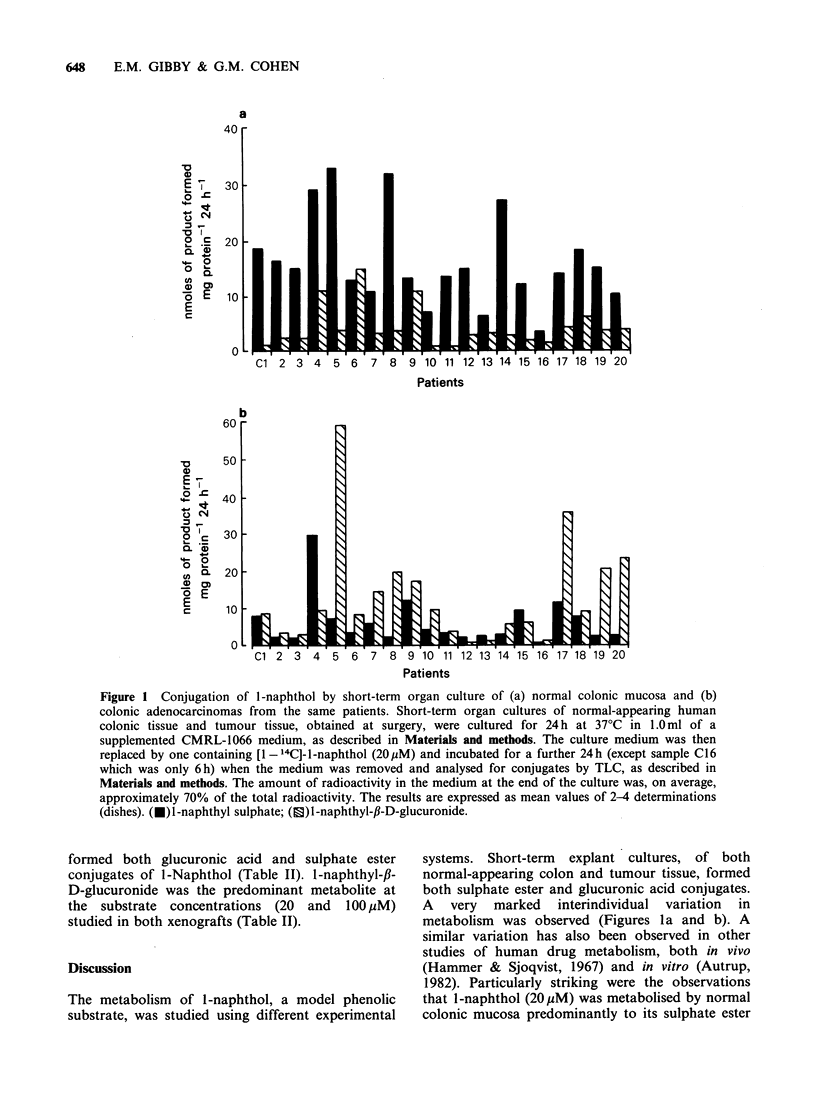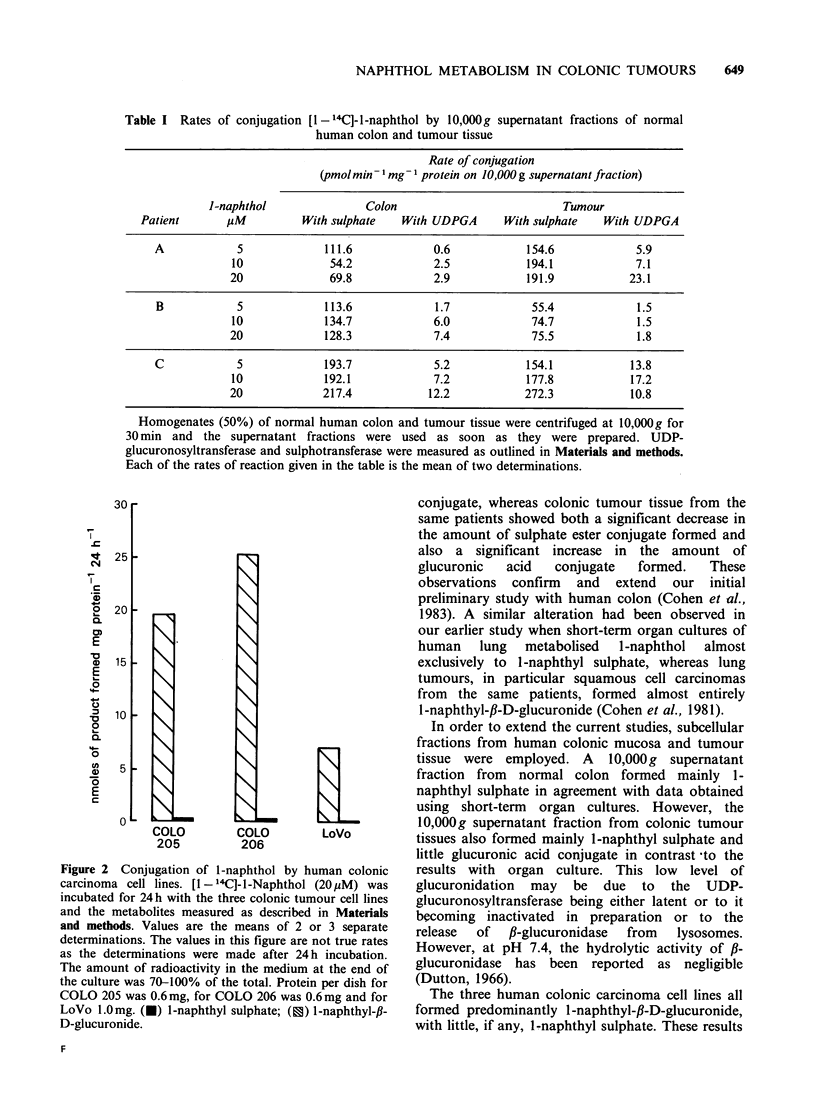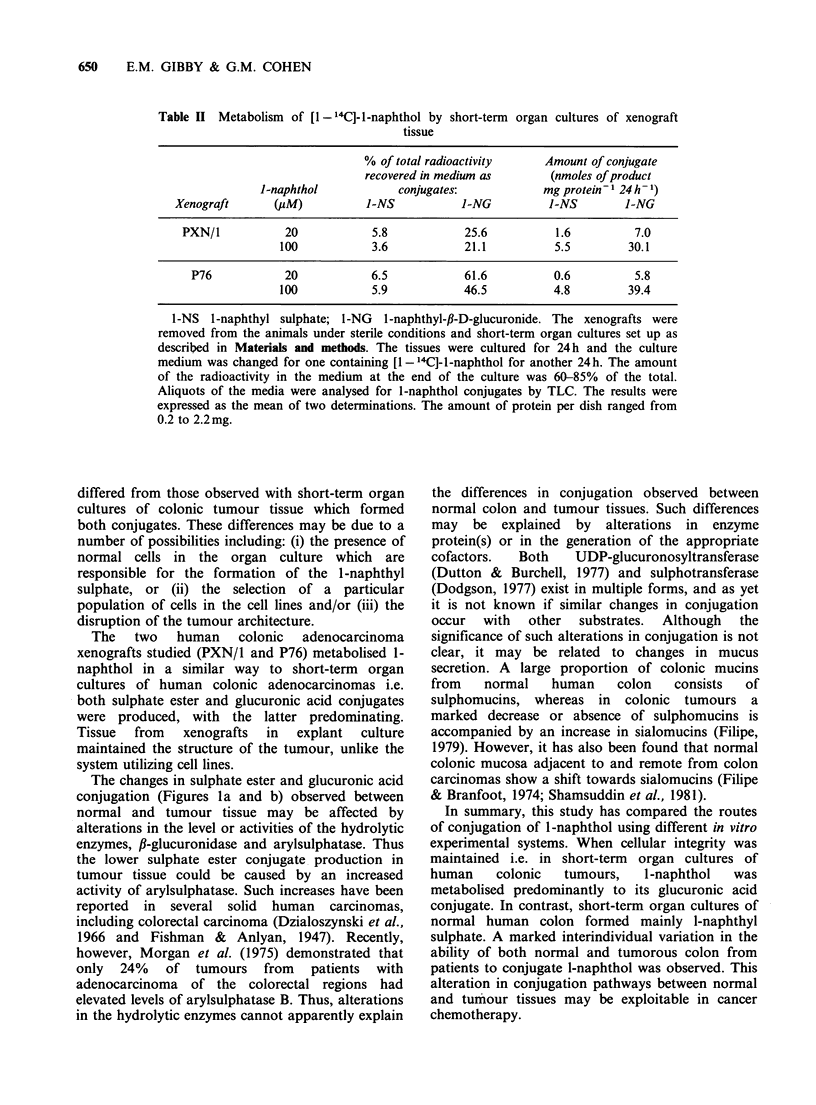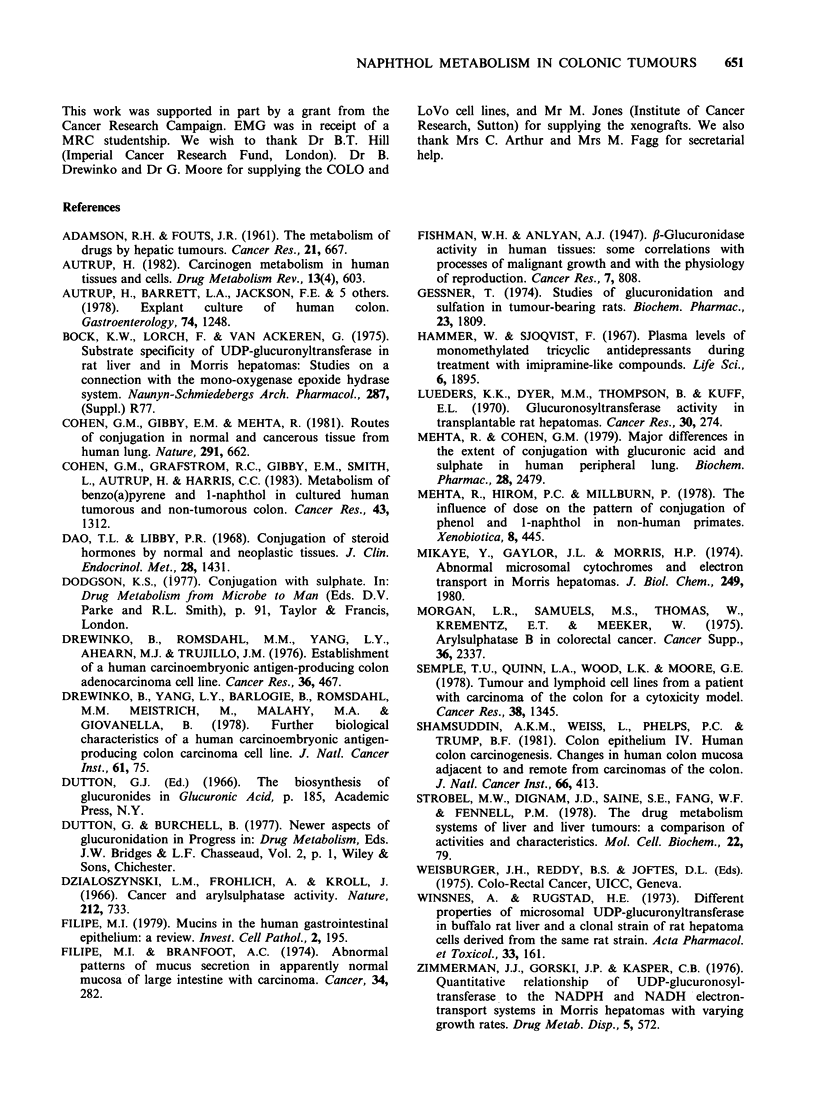# Conjugation of 1-naphthol by human colon and tumour tissue using different experimental systems.

**DOI:** 10.1038/bjc.1984.100

**Published:** 1984-05

**Authors:** E. M. Gibby, G. M. Cohen

## Abstract

The metabolism of 1-naphthol, a model phenolic substrate, to its glucuronic acid and sulphate ester conjugates has been studied in short-term organ cultures of normal human colon and tumour tissue, subcellular fractions of these tissues, human colonic tumour cell lines and human colonic tumour xenografts. Normal colonic tissue, in short-term organ culture, formed more 1-naphthyl sulphate than glucuronic acid conjugates. In contrast the colonic tumours, under the same conditions, produced more 1-naphthyl beta-D-glucuronide than 1-naphthyl sulphate. A marked interindividual variation in sulphate ester and glucuronic acid conjugation was noted in both normal and tumorous colon. The conjugation of 1-naphthol was also investigated, using subcellular fractions, where the metabolism found with normal colon reflected that observed utilizing short-term organ culture, but that from colonic tumour samples did not. Cell lines derived from human colonic adenocarcinomas metabolised 1-naphthol almost exclusively to its glucuronic acid conjugate. Xenografts derived from human colonic tumours formed similar conjugates to surgical samples in culture. Thus somewhat different results were obtained dependent on the experimental model chosen. However, in all colonic tumour systems studied, when the cells remained intact and where tissue architecture was maintained, 1-naphthol was metabolised predominantly to its glucuronic acid conjugate.


*Br. J. Cancer* (1984), **49**, 645–651

### Conjugation of 1-naphthol by human colon and tumour tissue using different experimental systems

E.M. Gibby* & G.M. Cohen

*Toxicology Unit, Department of Pharmacology, 29/39 Brunswick Square, The School of Pharmacy, University of London, London WC1N 1AX, U.K.*

**Summary** The metabolism of 1-naphthol, a model phenolic substrate, to its glucuronic acid and sulphate ester conjugates has been studied in short-term organ cultures of normal human colon and tumour tissue, subcellular fractions of these tissues, human colonic tumour cell lines and human colonic tumour xenografts. Normal colonic tissue, in short-term organ culture, formed more 1-naphthyl sulphate than glucuronic acid conjugates. In contrast the colonic tumours, under the same conditions, produced more 1-naphthyl β-D-glucuronide than 1-naphthyl sulphate. A marked interindividual variation in sulphate ester and glucuronic acid conjugation was noted in both normal and tumorous colon. The conjugation of 1-naphthol was also investigated, using subcellular fractions, where the metabolism found with normal colon reflected that observed utilizing short-term organ culture, but that from colonic tumour samples did not. Cell lines derived from human colonic adenocarcinomas metabolised 1-naphthol almost exclusively to its glucuronic acid conjugate. Xenografts derived from human colonic tumours formed similar conjugates to surgical samples in culture. Thus somewhat different results were obtained dependent on the experimental model chosen. However, in all colonic tumour systems studied, when the cells remained intact and where tissue architecture was maintained, 1-naphthol was metabolised predominantly to its glucuronic acid conjugate.

Colon cancer is a major disease in the Western World. It has a poor prognosis and treatment with chemotherapy has been disappointing (Weisburger *et al.*, 1975). A better understanding of the xenobiotic metabolising enzymes present in normal human colon and colonic tumours may aid in the identification of biochemical differences which may be exploitable for chemotherapy.

Several different experimental models are available to study the pathways of drug metabolism with human tissues. Such tissues may be obtained at surgery or autopsy and cultured *in vitro*. Classical biochemical subcellular fractionation can be used to study drug metabolism. However, in this investigation, greater reliance is placed on experiments which have maintained both cellular integrity and tissue architecture, i.e. explant organ culture. Another approach is to use cell lines established from human tumours. Human tumours can also be maintained as xenografts in mice whose immunological responses are depressed. Cell suspensions or cultures and explant organ culture, i.e., intact cellular systems, have advantages in that the full complement of drug-metabolising enzymes is present and possible rate limiting phenomena such as cofactor levels, control mechanisms and cellular transport systems are preserved as found *in vivo*. However, studies with subcellular fractions are simpler to perform and the results give an indication of the presence or absence of enzymes, but not whether such reactions occur *in vivo*.

The majority of studies on the xenobiotic metabolising enzymes in tumour tissues have concentrated on Phase I oxidative reactions, most commonly in rodent hepatomas of different growth rates (Adamson & Fouts, 1961; Miyake *et al.*, 1974; Strobel *et al.*, 1978; Zimmerman *et al.*, 1976). Other investigations have studied the Phase II conjugation reactions of drug metabolising enzymes. Various rodent hepatomas have increased UDP-glucuronosyltransferase activity when compared with the corresponding normal tissue using various substrates, including 1-naphthol, *p*-nitrophenol and *o*- and *p*-aminophenol (Zimmerman *et al.*, 1976; Lueders *et al.*, 1970; Winsnes & Rugstad, 1973; Gessner, 1974; Bock *et al.*, 1975). In the Reuber H-35 hepatoma, this increase in glucuronic acid conjugation was accompanied by a greatly decreased phenol sulphotransferase activity to *p*-nitrophenol (Gessner, 1974). Human mammary neoplasms have a variable pattern of steroid-sulphating activity which differ from either normal breast tissue or normal liver (Dao & Libby, 1968).

Previous results in our laboratory using short-term organ cultures of normal human lung have shown that 1-naphthol is metabolised primarily to sulphate ester conjugates (Mehta & Cohen, 1979), whereas tumour tissue from the same patients, in particular those with squamous cell carcinomas, formed almost exclusively the glucuronic acid

Correspondence: G.M. Cohen.

*Present address: Laboratory of Cellular Chemotherapy, Imperial Cancer Research Fund, Lincoln's Inn Fields, London WC2A 3PX, UK.

Received 17 November 1983; accepted 7 February 1984.

© The Macmillan Press Ltd.

conjugate (Cohen *et al.*, 1981). Thus, a biochemical difference is present which may be exploitable in cancer chemotherapy.

In the present study, the conjugating ability of normal human colon and colonic tumour tissue was investigated in more than one experimental system. In intact cellular systems, colonic tumours or cells from such tumours formed more glucuronic acid and less sulphate ester conjugates than did normal colonic mucosa. The cell lines established from colonic tumours formed 1-naphthyl-$\beta$-D-glucuronide almost exclusively.

### Materials and methods

### *Human surgical samples*

Normal human colon and tumour tissue were obtained at the time of surgery and transported to the laboratory in Leibovitz L-15 medium (Gibco Biocult, Paisley, Scotland) containing the antibiotics gentamicin sulphate ($50\,\mu g\,ml^{-1}$, Sigma), fungizone ($50\,\mu g\,ml^{-1}$, Gibco Biocult), penicillin ($100\,u\,ml^{-1}$) and streptomycin ($100\,\mu g\,ml^{-1}$, Gibco Biocult). The cancerous character of the tissues was established by histopathological examination. Short-term organ cultures of normal colon and tumour tissues were maintained essentially as previously described by Autrup *et al.*, 1978. Tissue explants on Gelfoam sponge, in petri dishes, were placed in a Bellco incubator (Bellco Glass Inc., New Jersey, USA), gassed with filtered 95% $O_2$ and 5% $CO_2$ and rocked at $10\,cycles\,min^{-1}$ for 24 h so that the explants were in the medium for 50% of each cycle.

### *Cell lines*

*COLO 205, COLO 206, LoVo* The three cell lines were all derived from human colonic adenocarcinomas and kindly supplied by Dr B.T. Hill (Imperial Cancer Research Fund, London, England). The cell lines COLO 205 and COLO 206, established by Semple *et al.*, 1978, were cultured in RPMI 1640 containing 10% bovine foetal calf serum, penicillin ($100\,u\,ml^{-1}$) and streptomycin ($100\,\mu g\,ml^{-1}$), in plastic tissue culture flasks (Sterilin Ltd., London, England) maintained at 37° in an atmosphere of 5% carbon dioxide in air. The LoVo cell line, established by Drewinko *et al.* (1976, 1978) was cultured in Hams medium containing 10% bovine foetal calf serum, glutamine (1 mM), penicillin ($100\,u\,ml^{-1}$) and streptomycin ($100\,\mu g,ml^{-1}$) in plastic tissue culture flasks (Nunc, Denmark) maintained in an atmosphere of 5% carbon dioxide in air at 37°C.

### *Xenografts*

Nude athymic mice with implanted human colonic adenocarcinoma xenografts (PXN/1 and P76) were kindly provided by Mr M. Jones, Institute of Cancer Research (Sutton, Surrey, England). The xenograft tissue was removed from the mice and cultured as short-term explants in the same way as the human tissue from surgery.

### *Metabolism of [1 − $^{14}C$]-1-naphthol by short-term explant culture of colonic surgical samples and xenografts*

After culture at 37°C for a period of 24 h, the medium was decanted and replaced with one containing [1 − $^{14}C$]-1-naphthol (Amersham International Ltd., Bucks., U.K. Sp. Act. $19.4\,mCi\,mmol^{-1}$) at various concentrations, multiple incubations being carried out. The tissue was then cultured for varying periods, after which the medium and tissue were removed and stored separately at −20°C, prior to analysis of 1-naphthol metabolites. In some cases, the tissue was cultured still further by replacing the media containing [1 − $^{14}C$]-1-naphthol with fresh culture media. Appropriate controls for the metabolism studies were obtained by incubating media containing [1 − $^{14}C$]-1-naphthol for 24 h in the absence of tissue and the media removed, stored and analysed. The tissue was dissolved in 100 $\mu$l of 1 M sodium hydroxide in a sealed tube at 37°C. Aliquots of the resulting solution were used for protein determination by the Lowry method.

### *Metabolism of [1 − $^{14}C$]-1-naphthol by colonic cell lines*

Fresh medium containing different concentrations of [1 − $^{14}C$]-1-naphthol was incubated with the cells at 37°C for 24 h. The medium was then decanted and stored frozen until analysis. The cells from each incubation were lysed by the addition of 0.1 M sodium hydroxide and protein determined (Lowry *et al.*, 1951).

### *Hydrolysis of conjugates for further identification of metabolites*

Aliquots of culture media were incubated with either $\beta$-glucuronidase ($5000\,u\,ml^{-1}$) in 0.1 M acetate buffer, pH 5.0, or arylsulphatase ($700\,u\,ml^{-1}$) and D-saccharic acid 1,4-lactone (40 mM) in 0.1 M acetate buffer, pH 5.0, or with 0.1 M acetate buffer, pH 5.0 alone. Saccharic acid 1,4-lactone was included with arylsulphatase to inhibit $\beta$-glucuronidase, a known contaminant of arylsulphatase. The hydrolyses were carried out in sealed tubes in a 37° incubator for periods between 20 and 24 h. In order to quantitate the conjugates formed, the hydrolyses were then analysed by TLC as described below. A metabolite was identified as a glucuronide if it was hydrolysed by $\beta$-glucuronidase and as a sulphate ester if it was hydrolysed by arylsulphatase.

*1-Naphthol conjugation by subcellular fractions*

Homogenates (50%) of tissue in 1.15% potassium chloride were centrifuged at 10,000 *g* for 30 min and the supernatant fractions were used in enzyme assays. For glucuronic acid conjugation, the reaction mixture (total volume 500 $\mu$l) consisted of 0.02–0.2 $\mu$Ci [$1-{}^{14}$C]-1-naphthol (2–20 $\mu$M), 1.5 mM UDP-glucuronic acid (UDPGA) in 0.05 M Tris-HCl pH 7.4 containing 4 mM magnesium chloride. To measure sulphation, the reaction mixture (total volume 500 $\mu$l) consisted of [$1-{}^{14}$C]-1-naphthol (2–20 $\mu$M), 2 mM sodium sulphate, 5 mM magnesium chloride, 5 mM ATP and 0.05 M Tris-HCl pH 7.4. The reactions were initiated by the addition of supernatant and incubated at 37°C in a shaking water bath for periods of time between 10 to 60 min in order to ensure linearity of the reaction. Control incubations contained no enzyme. Reactions were terminated by the removal of 100 $\mu$l aliquots of the incubation media into tubes with an equal volume of methanol containing the unlabelled standards 1-naphthol, 1-naphthyl-$\beta$-D-glucuronide and 1-naphthyl sulphate.

*Chromatography of 1-naphthol conjugates*

The conjugates in both the culture and incubation media were analysed by thin layer chromatography (TLC) essentially as previously described (Mehta *et al.*, 1978; Cohen *et al.*, 1981).

### Results

*Short-term organ cultures*

Short-term organ cultures of normal human colon metabolised 1-naphthol to both its sulphate ester and glucuronic acid conjugates. At low concentrations of 1-naphthol (20 $\mu$M), normal colon formed significantly more 1-naphthyl sulphate than 1-naphthyl-$\beta$-D-glucuronide (Figure 1a) ($P=0.005$). Considerable interindividual variation in the amounts of both conjugates formed was observed, ranging from 3.8–32.8 and 0.8–14.9 nmoles formed mg protein$^{-1}$ 24 h$^{-1}$ for 1-naphthyl sulphate and 1-naphthyl-$\beta$-D-glucuronide respectively. At higher concentrations of 1-naphthol (100 $\mu$M), the normal colon still formed significantly ($P=0.05$) more sulphate ester than glucuronic acid conjugates, although at this concentration, glucuronic acid conjugates represented a higher percentage of the total metabolites formed (results not shown).

Short-term organ cultures of colonic tumour tissue from the same patients also metabolised 1-naphthol (20 $\mu$M) to both sulphate ester and glucuronic acid conjugates (Figure 1b). The most striking observation was the decrease in sulphate ester conjugate formed by the tumour tissue compared to the normal (compare Figures 1a and b). The amount of 1-naphthyl-$\beta$-D-glucuronide formed by the tumour tissue was significantly greater than with normal colonic tissue ($P=0.025$). However, the overall metabolism of 1-naphthol to conjugates was lower in the tumour tissue compared to the normal ($P=0.01$) because the decrease in 1-naphthyl sulphate formation was greater than the increase in 1-naphthyl-$\beta$-D-glucuronide.

The above results describe the metabolites of 1-naphthol present in the culture media. At low concentrations of 1-naphthol (20 $\mu$M) the percentage of radioactivity trapped in the tissue explants was very small (<2%) and the metabolites associated with the tissues were similar to those seen in the medium.

*Subcellular fractions*

Supernatant fractions (10,000 *g*) of both normal colon and tumour tissues were used to measure rates of conjugation of 1-naphthol with sulphate and UDPGA (Table I). Both normal and tumour tissue formed significantly more sulphate ester than glucuronic acid conjugates, and with both tissues only very small amounts of the latter metabolite were formed (Table I). A concentration dependent increase in conjugate formation was generally observed (Table I). Incubations with rat liver 10,000 *g* supernatant fractions, run in parallel in each case, showed over 90% conversion to the glucuronide with 1-naphthol, indicating that the incubation conditions were satisfactory (results not shown).

*Human colonic carcinoma cell lines*

All three human colonic carcinoma cell lines produced 1-naphthyl-$\beta$-D-glucuronide with little, if any, 1-naphthyl sulphate being formed (Figure 2). When the concentration of 1-naphthol was increased from 5–20 $\mu$M, all three cell lines showed an increased production of 1-naphthyl-$\beta$-D-glucuronide. Above this concentration, the formation of the glucuronic acid conjugate decreased, possibly due to toxicity of 1-naphthol at these higher concentrations or to substrate inhibition (results not shown).

*Xenografts*

Short-term organ cultures of two human colonic adenocarcinoma xenografts (PXN/1 and P76)

**Figure 1** Conjugation of 1-naphthol by short-term organ culture of (a) normal colonic mucosa and (b) colonic adenocarcinomas from the same patients. Short-term organ cultures of normal-appearing human colonic tissue and tumour tissue, obtained at surgery, were cultured for 24 h at 37°C in 1.0 ml of a supplemented CMRL-1066 medium, as described in **Materials and methods**. The culture medium was then replaced by one containing [1 – $^{14}$C]-1-naphthol (20 $\mu$M) and incubated for a further 24 h (except sample C16 which was only 6 h) when the medium was removed and analysed for conjugates by TLC, as described in **Materials and methods**. The amount of radioactivity in the medium at the end of the culture was, on average, approximately 70% of the total radioactivity. The results are expressed as mean values of 2–4 determinations (dishes). (■)1-naphthyl sulphate; (▨)1-naphthyl-$\beta$-D-glucuronide.

formed both glucuronic acid and sulphate ester conjugates of 1-Naphthol (Table II). 1-naphthyl-$\beta$-D-glucuronide was the predominant metabolite at the substrate concentrations (20 and 100 $\mu$M) studied in both xenografts (Table II).

### Discussion

The metabolism of 1-naphthol, a model phenolic substrate, was studied using different experimental systems. Short-term explant cultures, of both normal-appearing colon and tumour tissue, formed both sulphate ester and glucuronic acid conjugates. A very marked interindividual variation in metabolism was observed (Figures 1a and b). A similar variation has also been observed in other studies of human drug metabolism, both *in vivo* (Hammer & Sjoqvist, 1967) and *in vitro* (Autrup, 1982). Particularly striking were the observations that 1-naphthol (20 $\mu$M) was metabolised by normal colonic mucosa predominantly to its sulphate ester

**Table I** Rates of conjugation [1 – $^{14}$C]-1-naphthol by 10,000*g* supernatant fractions of normal human colon and tumour tissue

| | | *Rate of conjugation (pmol min$^{-1}$ mg$^{-1}$ protein on 10,000 g supernatant fraction)* | | | |
|---|---|---|---|---|---|
| | | *Colon* | | *Tumour* | |
| *Patient* | *1-naphthol μM* | *With sulphate* | *With UDPGA* | *With sulphate* | *With UDPGA* |
| A | 5 | 111.6 | 0.6 | 154.6 | 5.9 |
| | 10 | 54.2 | 2.5 | 194.1 | 7.1 |
| | 20 | 69.8 | 2.9 | 191.9 | 23.1 |
| B | 5 | 113.6 | 1.7 | 55.4 | 1.5 |
| | 10 | 134.7 | 6.0 | 74.7 | 1.5 |
| | 20 | 128.3 | 7.4 | 75.5 | 1.8 |
| C | 5 | 193.7 | 5.2 | 154.1 | 13.8 |
| | 10 | 192.1 | 7.2 | 177.8 | 17.2 |
| | 20 | 217.4 | 12.2 | 272.3 | 10.8 |

Homogenates (50%) of normal human colon and tumour tissue were centrifuged at 10,000*g* for 30 min and the supernatant fractions were used as soon as they were prepared. UDP-glucuronosyltransferase and sulphotransferase were measured as outlined in **Materials and methods.** Each of the rates of reaction given in the table is the mean of two determinations.

**Figure 2** Conjugation of 1-naphthol by human colonic carcinoma cell lines. [1 – $^{14}$C]-1-Naphthol (20 μM) was incubated for 24 h with the three colonic tumour cell lines and the metabolites measured as described in **Materials and methods**. Values are the means of 2 or 3 separate determinations. The values in this figure are not true rates as the determinations were made after 24 h incubation. The amount of radioactivity in the medium at the end of the culture was 70–100% of the total. Protein per dish for COLO 205 was 0.6 mg, for COLO 206 was 0.6 mg and for LoVo 1.0 mg. (■) 1-naphthyl sulphate; (▧) 1-naphthyl-β-D-glucuronide.

conjugate, whereas colonic tumour tissue from the same patients showed both a significant decrease in the amount of sulphate ester conjugate formed and also a significant increase in the amount of glucuronic acid conjugate formed. These observations confirm and extend our initial preliminary study with human colon (Cohen *et al.*, 1983). A similar alteration had been observed in our earlier study when short-term organ cultures of human lung metabolised 1-naphthol almost exclusively to 1-naphthyl sulphate, whereas lung tumours, in particular squamous cell carcinomas from the same patients, formed almost entirely 1-naphthyl-β-D-glucuronide (Cohen *et al.*, 1981).

In order to extend the current studies, subcellular fractions from human colonic mucosa and tumour tissue were employed. A 10,000*g* supernatant fraction from normal colon formed mainly 1-naphthyl sulphate in agreement with data obtained using short-term organ cultures. However, the 10,000*g* supernatant fraction from colonic tumour tissues also formed mainly 1-naphthyl sulphate and little glucuronic acid conjugate in contrast to the results with organ culture. This low level of glucuronidation may be due to the UDP-glucuronosyltransferase being either latent or to it becoming inactivated in preparation or to the release of β-glucuronidase from lysosomes. However, at pH 7.4, the hydrolytic activity of β-glucuronidase has been reported as negligible (Dutton, 1966).

The three human colonic carcinoma cell lines all formed predominantly 1-naphthyl-β-D-glucuronide, with little, if any, 1-naphthyl sulphate. These results

**Table II** Metabolism of $[1-{}^{14}C]$-1-naphthol by short-term organ cultures of xenograft tissue

| Xenograft | *1-naphthol* (*μM*) | *% of total radioactivity recovered in medium as conjugates*: *1-NS* | *1-NG* | *Amount of conjugate (nmoles of product mg protein$^{-1}$ 24 h$^{-1}$)* *1-NS* | *1-NG* |
|---|---|---|---|---|---|
| PXN/1 | 20 | 5.8 | 25.6 | 1.6 | 7.0 |
| | 100 | 3.6 | 21.1 | 5.5 | 30.1 |
| P76 | 20 | 6.5 | 61.6 | 0.6 | 5.8 |
| | 100 | 5.9 | 46.5 | 4.8 | 39.4 |

1-NS 1-naphthyl sulphate; 1-NG 1-naphthyl-β-D-glucuronide. The xenografts were removed from the animals under sterile conditions and short-term organ cultures set up as described in **Materials and methods**. The tissues were cultured for 24 h and the culture medium was changed for one containing $[1-{}^{14}C]$-1-naphthol for another 24 h. The amount of the radioactivity in the medium at the end of the culture was 60–85% of the total. Aliquots of the media were analysed for 1-naphthol conjugates by TLC. The results were expressed as the mean of two determinations. The amount of protein per dish ranged from 0.2 to 2.2 mg.

differed from those observed with short-term organ cultures of colonic tumour tissue which formed both conjugates. These differences may be due to a number of possibilities including: (i) the presence of normal cells in the organ culture which are responsible for the formation of the 1-naphthyl sulphate, or (ii) the selection of a particular population of cells in the cell lines and/or (iii) the disruption of the tumour architecture.

The two human colonic adenocarcinoma xenografts studied (PXN/1 and P76) metabolised 1-naphthol in a similar way to short-term organ cultures of human colonic adenocarcinomas i.e. both sulphate ester and glucuronic acid conjugates were produced, with the latter predominating. Tissue from xenografts in explant culture maintained the structure of the tumour, unlike the system utilizing cell lines.

The changes in sulphate ester and glucuronic acid conjugation (Figures 1a and b) observed between normal and tumour tissue may be affected by alterations in the level or activities of the hydrolytic enzymes, β-glucuronidase and arylsulphatase. Thus the lower sulphate ester conjugate production in tumour tissue could be caused by an increased activity of arylsulphatase. Such increases have been reported in several solid human carcinomas, including colorectal carcinoma (Dzialoszynski *et al.*, 1966 and Fishman & Anlyan, 1947). Recently, however, Morgan *et al.* (1975) demonstrated that only 24% of tumours from patients with adenocarcinoma of the colorectal regions had elevated levels of arylsulphatase B. Thus, alterations in the hydrolytic enzymes cannot apparently explain the differences in conjugation observed between normal colon and tumour tissues. Such differences may be explained by alterations in enzyme protein(s) or in the generation of the appropriate cofactors. Both UDP-glucuronosyltransferase (Dutton & Burchell, 1977) and sulphotransferase (Dodgson, 1977) exist in multiple forms, and as yet it is not known if similar changes in conjugation occur with other substrates. Although the significance of such alterations in conjugation is not clear, it may be related to changes in mucus secretion. A large proportion of colonic mucins from normal human colon consists of sulphomucins, whereas in colonic tumours a marked decrease or absence of sulphomucins is accompanied by an increase in sialomucins (Filipe, 1979). However, it has also been found that normal colonic mucosa adjacent to and remote from colon carcinomas show a shift towards sialomucins (Filipe & Branfoot, 1974; Shamsuddin *et al.*, 1981).

In summary, this study has compared the routes of conjugation of 1-naphthol using different *in vitro* experimental systems. When cellular integrity was maintained i.e. in short-term organ cultures of human colonic tumours, 1-naphthol was metabolised predominantly to its glucuronic acid conjugate. In contrast, short-term organ cultures of normal human colon formed mainly 1-naphthyl sulphate. A marked interindividual variation in the ability of both normal and tumorous colon from patients to conjugate 1-naphthol was observed. This alteration in conjugation pathways between normal and tumour tissues may be exploitable in cancer chemotherapy.

This work was supported in part by a grant from the Cancer Research Campaign. EMG was in receipt of a MRC studentship. We wish to thank Dr B.T. Hill (Imperial Cancer Research Fund, London). Dr B. Drewinko and Dr G. Moore for supplying the COLO and LoVo cell lines, and Mr M. Jones (Institute of Cancer Research, Sutton) for supplying the xenografts. We also thank Mrs C. Arthur and Mrs M. Fagg for secretarial help.

### References

ADAMSON, R.H. & FOUTS, J.R. (1961). The metabolism of drugs by hepatic tumours. *Cancer Res.*, **21**, 667.

AUTRUP, H. (1982). Carcinogen metabolism in human tissues and cells. *Drug Metabolism Rev.*, **13**(4), 603.

AUTRUP, H., BARRETT, L.A., JACKSON, F.E. & 5 others. (1978). Explant culture of human colon. *Gastroenterology*, **74**, 1248.

BOCK, K.W., LORCH, F. & VAN ACKEREN, G. (1975). Substrate specificity of UDP-glucuronyltransferase in rat liver and in Morris hepatomas: Studies on a connection with the mono-oxygenase epoxide hydrase system. *Naunyn-Schmiedebergs Arch. Pharmacol.*, **287**, (Suppl.) R77.

COHEN, G.M., GIBBY, E.M. & MEHTA, R. (1981). Routes of conjugation in normal and cancerous tissue from human lung. *Nature*, **291**, 662.

COHEN, G.M., GRAFSTROM, R.C., GIBBY, E.M., SMITH, L., AUTRUP, H. & HARRIS, C.C. (1983). Metabolism of benzo(a)pyrene and 1-naphthol in cultured human tumorous and non-tumorous colon. *Cancer Res.*, **43**, 1312.

DAO, T.L. & LIBBY, P.R. (1968). Conjugation of steroid hormones by normal and neoplastic tissues. *J. Clin. Endocrinol. Met.*, **28**, 1431.

DODGSON, K.S., (1977). Conjugation with sulphate. In: *Drug Metabolism from Microbe to Man* (Eds. D.V. Parke and R.L. Smith), p. 91, Taylor & Francis, London.

DREWINKO, B., ROMSDAHL, M.M., YANG, L.Y., AHEARN, M.J. & TRUJILLO, J.M. (1976). Establishment of a human carcinoembryonic antigen-producing colon adenocarcinoma cell line. *Cancer Res.*, **36**, 467.

DREWINKO, B., YANG, L.Y., BARLOGIE, B., ROMSDAHL, M.M. MEISTRICH, M., MALAHY, M.A. & GIOVANELLA, B. (1978). Further biological characteristics of a human carcinoembryonic antigen-producing colon carcinoma cell line. *J. Natl. Cancer Inst.*, **61**, 75.

DUTTON, G.J. (Ed.) (1966). The biosynthesis of glucuronides in *Glucuronic Acid*, p. 185, Academic Press, N.Y.

DUTTON, G. & BURCHELL, B. (1977). Newer aspects of glucuronidation in Progress in: *Drug Metabolism*, Eds. J.W. Bridges & L.F. Chasseaud, Vol. 2, p. 1, Wiley & Sons, Chichester.

DZIALOSZYNSKI, L.M., FROHLICH, A. & KROLL, J. (1966). Cancer and arylsulphatase activity. *Nature*, **212**, 733.

FILIPE, M.I. (1979). Mucins in the human gastrointestinal epithelium: a review. *Invest. Cell Pathol.*, **2**, 195.

FILIPE, M.I. & BRANFOOT, A.C. (1974). Abnormal patterns of mucus secretion in apparently normal mucosa of large intestine with carcinoma. *Cancer*, **34**, 282.

FISHMAN, W.H. & ANLYAN, A.J. (1947). β-Glucuronidase activity in human tissues: some correlations with processes of malignant growth and with the physiology of reproduction. *Cancer Res.*, **7**, 808.

GESSNER, T. (1974). Studies of glucuronidation and sulfation in tumour-bearing rats. *Biochem. Pharmac.*, **23**, 1809.

HAMMER, W. & SJOQVIST, F. (1967). Plasma levels of monomethylated tricyclic antidepressants during treatment with imipramine-like compounds. *Life Sci.*, **6**, 1895.

LUEDERS, K.K., DYER, M.M., THOMPSON, B. & KUFF, E.L. (1970). Glucuronosyltransferase activity in transplantable rat hepatomas. *Cancer Res.*, **30**, 274.

MEHTA, R. & COHEN, G.M. (1979). Major differences in the extent of conjugation with glucuronic acid and sulphate in human peripheral lung. *Biochem. Pharmac.*, **28**, 2479.

MEHTA, R., HIROM, P.C. & MILLBURN, P. (1978). The influence of dose on the pattern of conjugation of phenol and 1-naphthol in non-human primates. *Xenobiotica*, **8**, 445.

MIKAYE, Y., GAYLOR, J.L. & MORRIS, H.P. (1974). Abnormal microsomal cytochromes and electron transport in Morris hepatomas. *J. Biol. Chem.*, **249**, 1980.

MORGAN, L.R., SAMUELS, M.S., THOMAS, W., KREMENTZ, E.T. & MEEKER, W. (1975). Arylsulphatase B in colorectal cancer. *Cancer* Supp., **36**, 2337.

SEMPLE, T.U., QUINN, L.A., WOOD, L.K. & MOORE, G.E. (1978). Tumour and lymphoid cell lines from a patient with carcinoma of the colon for a cytoxicity model. *Cancer Res.*, **38**, 1345.

SHAMSUDDIN, A.K.M., WEISS, L., PHELPS, P.C. & TRUMP, B.F. (1981). Colon epithelium IV. Human colon carcinogenesis. Changes in human colon mucosa adjacent to and remote from carcinomas of the colon. *J. Natl. Cancer Inst.*, **66**, 413.

STROBEL, M.W., DIGNAM, J.D., SAINE, S.E., FANG, W.F. & FENNELL, P.M. (1978). The drug metabolism systems of liver and liver tumours: a comparison of activities and characteristics. *Mol. Cell. Biochem.*, **22**, 79.

WEISBURGER, J.H., REDDY, B.S. & JOFTES, D.L. (Eds). (1975). Colo-Rectal Cancer, UICC, Geneva.

WINSNES, A. & RUGSTAD, H.E. (1973). Different properties of microsomal UDP-glucuronyltransferase in buffalo rat liver and a clonal strain of rat hepatoma cells derived from the same rat strain. *Acta Pharmacol. et Toxicol.*, **33**, 161.

ZIMMERMAN, J.J., GORSKI, J.P. & KASPER, C.B. (1976). Quantitative relationship of UDP-glucuronosyltransferase to the NADPH and NADH electron-transport systems in Morris hepatomas with varying growth rates. *Drug Metab. Disp.*, **5**, 572.